# Supplementary material for: Stability Assessment of p-i-n Perovskite Photovoltaic Mini-Modules Utilizing Different Top Metal Electrodes
Source: Micromachines (Basel). 2021 Apr 13;12(4):423. doi: 10.3390/mi12040423 (PMC8069674; doi:10.3390/mi12040423)
Supplement: Supplementary file 1 [file micromachines-12-00423-s001.pdf]

# Supporting Information

# Stability assessment of p-i-n perovskite photovoltaic mini-modules utilizing different top metal electrodes

Janardan Dagar[1a*], Gopinath Paramasivam[1a], Carola Klimm[1a], Markus Fenske[2,3], Christof Schultz[2], Rutger Schlatmann[2,3], Bert Stegemann[2], Eva Unger[1a, 4]

[1]Helmholtz-Zentrum Berlin, HySPRINT Innovation Lab, Kekuléstrasse 5, 12489 Berlin, Germany.

[a]Young Investigator Group Hybrid Materials Formation and Scaling Kekuléstrasse 5, 12489 Berlin, Germany.

[2]HTW Berlin – University of Applied Sciences, Wilhelminenhofstr. 75a, D-12459 Berlin, Germany.

[3]PVcomB / Helmholtz-Zentrum Berlin für Materialien und Energie GmbH, Schwarzschildstr. 3, D-12489 Berlin, Germany.

[4]Department of Chemistry & NanoLund, Lund University, Naturvetarvägen 14, 22362 Lund, Sweden.

**Corresponding Author**

*Janardan.Dagar@helmholtz-berlin.de

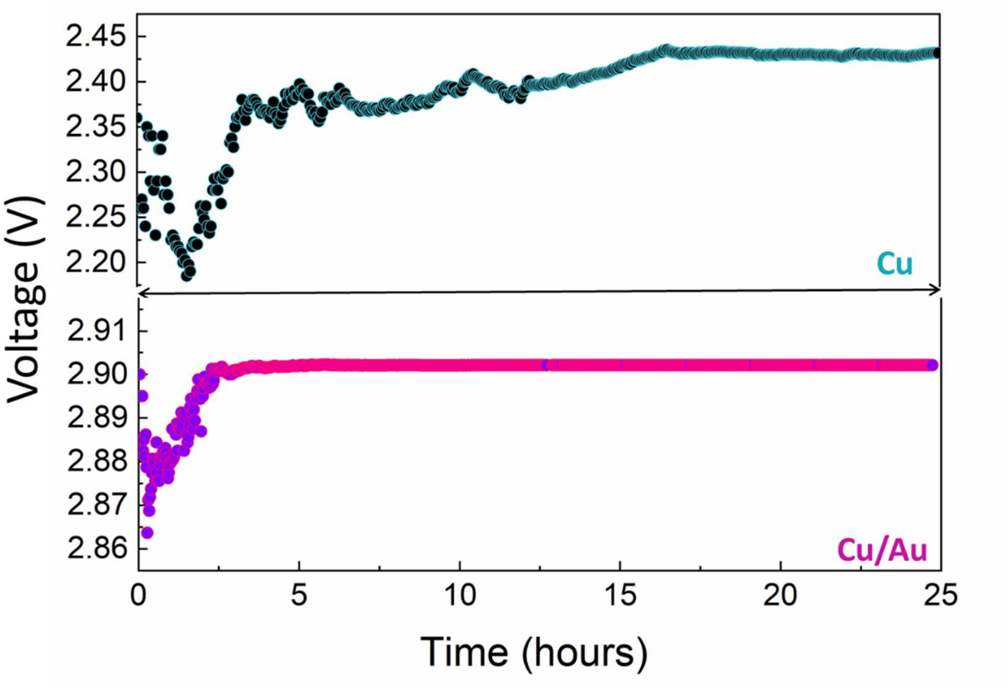


**Figure S1.** Steady state voltage ($V_{MPP}$) measured under 1 sun illumination for both Cu and Cu/Au based mini modules.

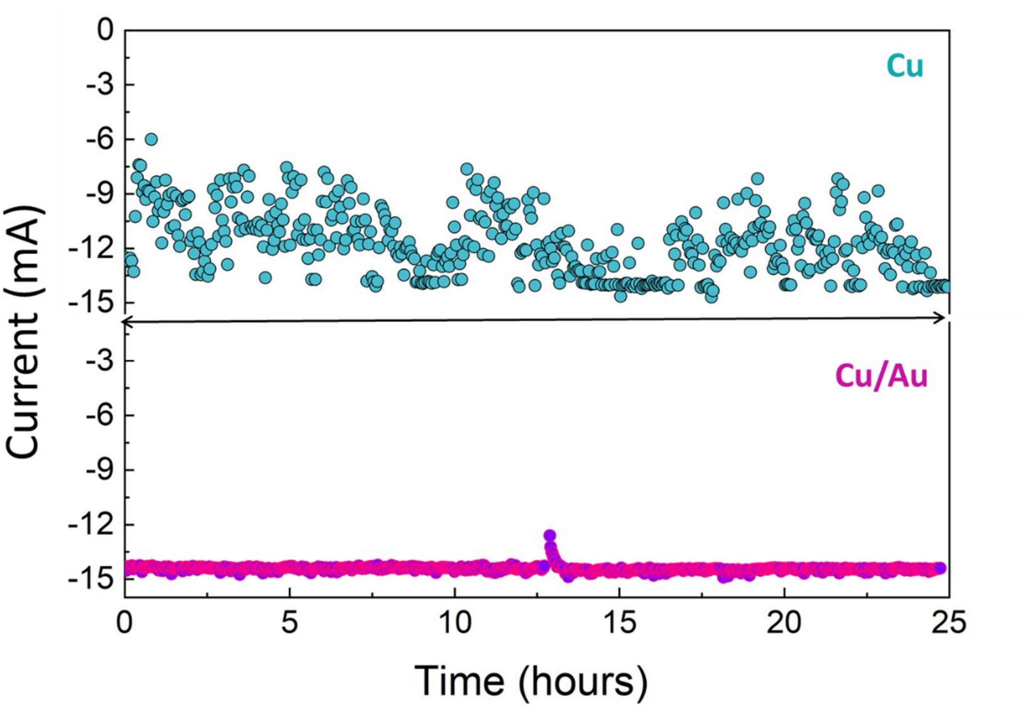


**Figure S2.** Steady state current ($I_{MPP}$) measured under 1 sun illumination for both Cu and Cu/Au based mini modules.

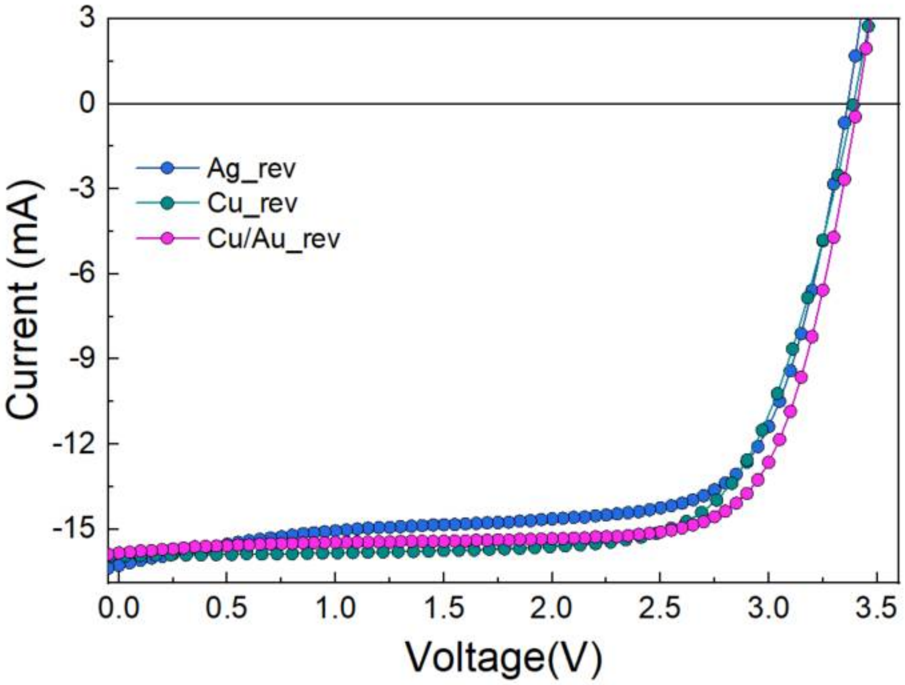


**Figure S3.** Current-voltage (*I-V*) measurements of best-performing perovskite solar mini-modules (ITO|2PACz|CsFAPb(Br, I)$_3$|$C_{60}$|$SnO_2$ |Ag or Cu or Cu/Au measured under simulated AM 1.5G illumination.

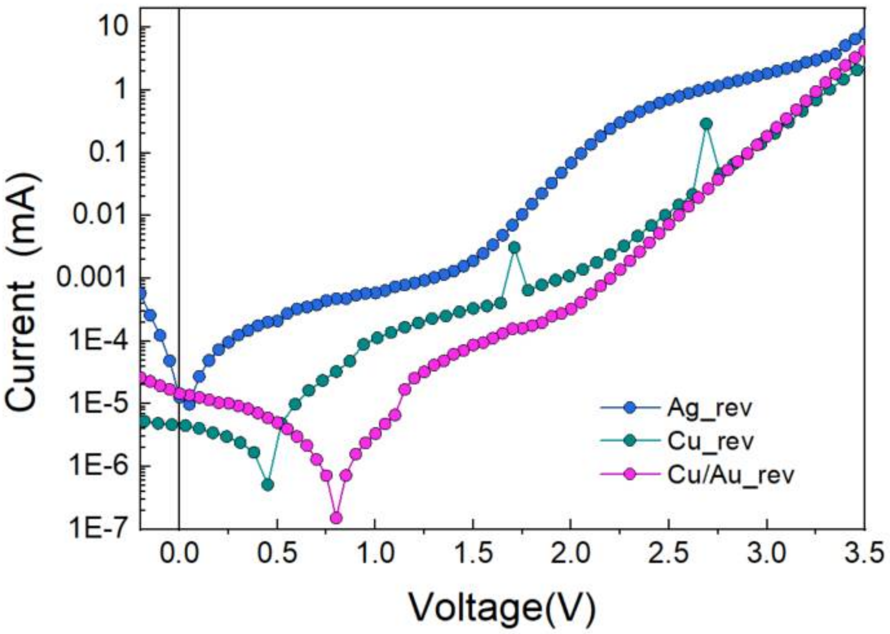


**Figure S4.** Current–voltage *(I–V)*-characteristics perovskite solar minimodules measured under dark.

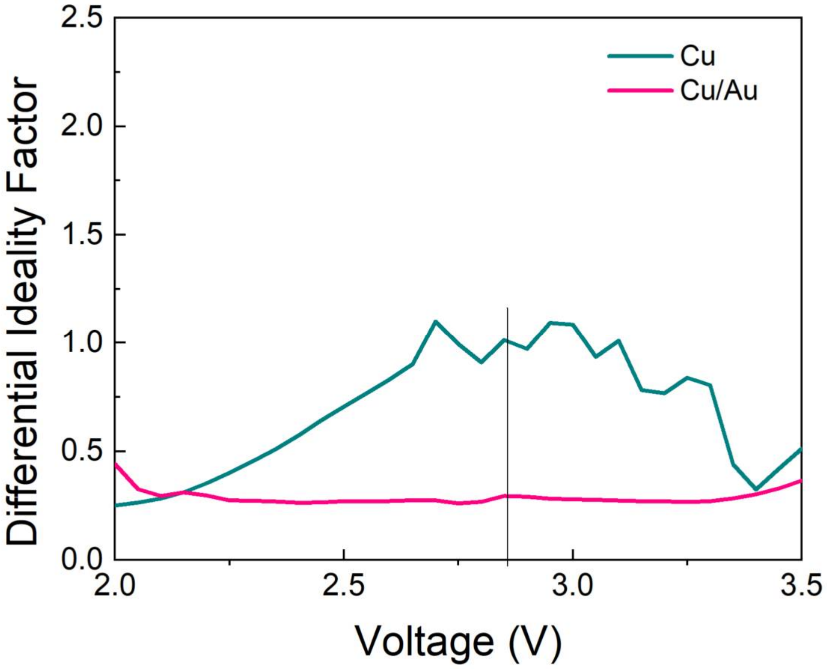


**Figure S5.** Ideality factors calculated from *I–V* curves measured under dark conditions for both Cu and Cu/Au based PSMs.

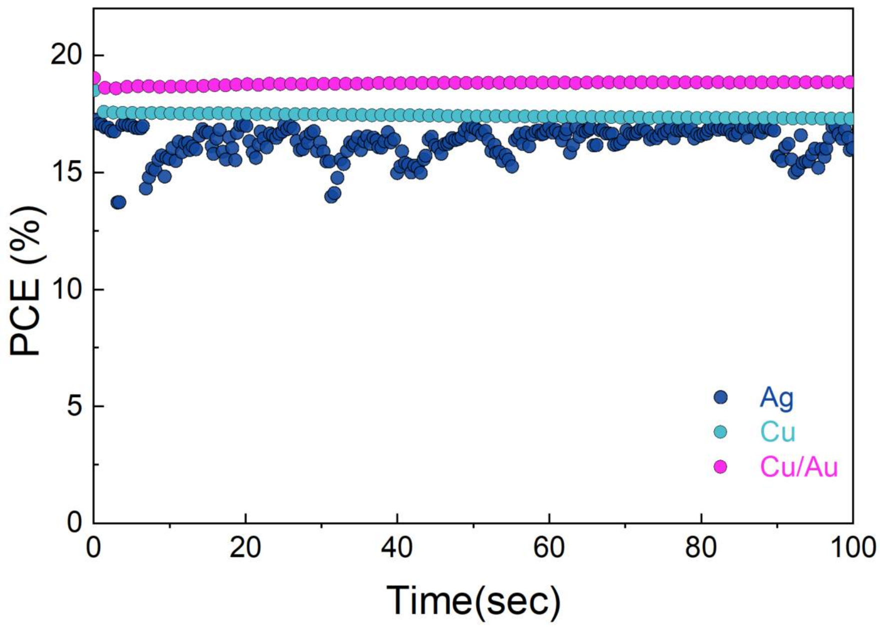


**Figure S6.** The maximum power point tracking (*MPPT*) measured for 100 s at the fixed voltage near the maximum power point (MPP) derived from *I-V* measurements.

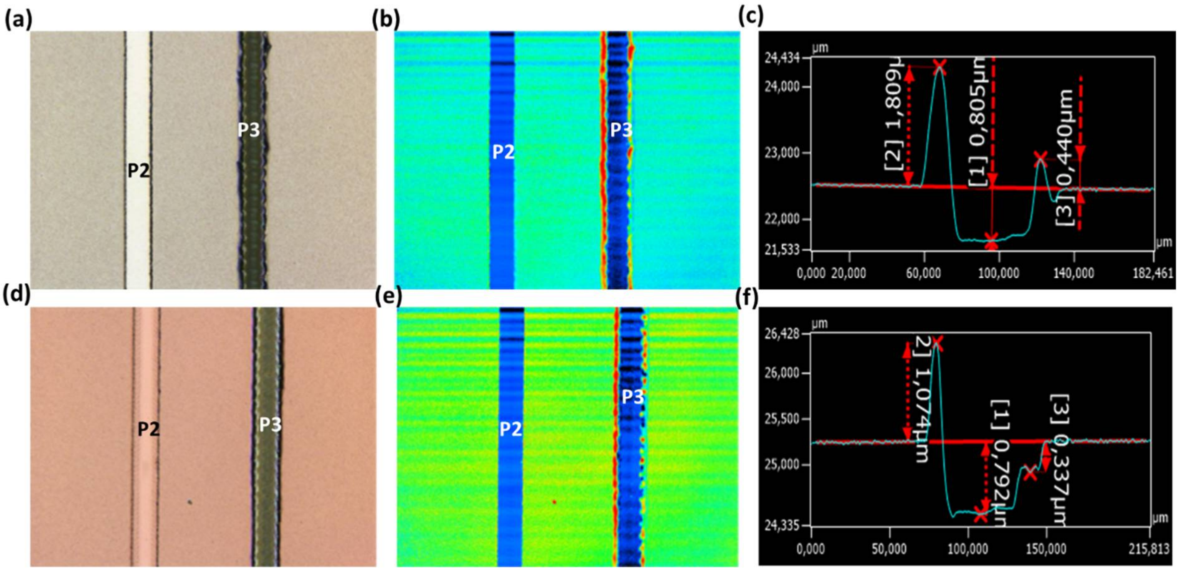


**Figure S7.** Optical microscopy images of P2 and P3 pattering of perovskite mini-module. (a-c) PSMs with Cu electrode, (d-f) with Cu/Au electrodes.

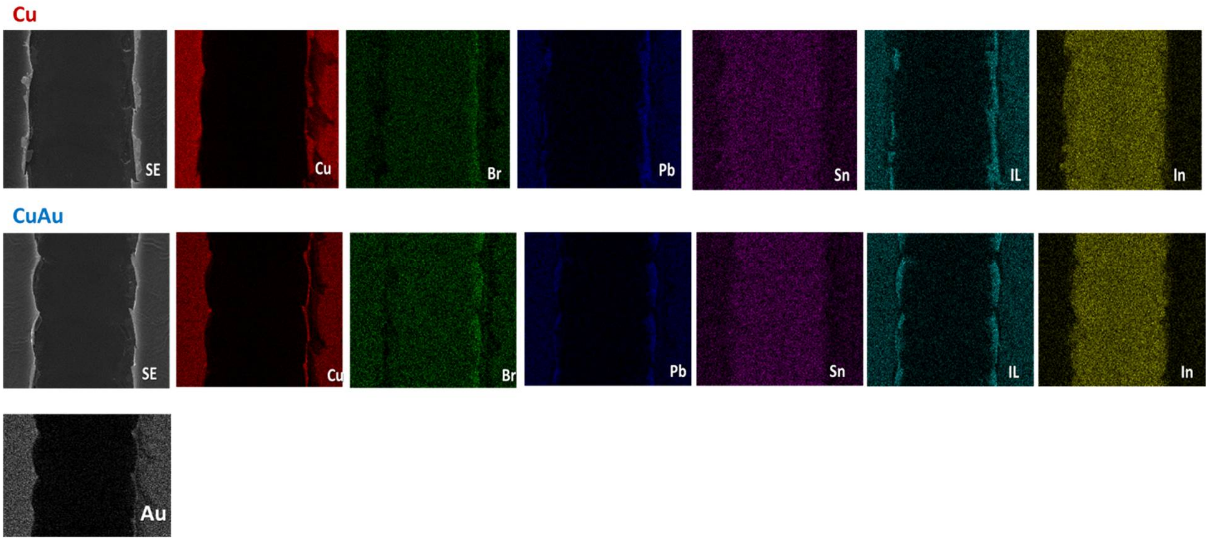


**Figure S8.** EDX images of P3 scribing of both Cu and Cu/Au based PSMs.
